# Biomechanical impact of discoid lateral meniscus and partial meniscectomy in the pediatric knee: a finite element study

**DOI:** 10.1007/s11517-025-03492-x

**Published:** 2026-01-14

**Authors:** Lourdes Segovia-García, Miryam B. Sánchez, María Teresa Carrascal-Morillo

**Affiliations:** Department of Mechanics, UNED, Madrid, Spain

**Keywords:** Discoid lateral meniscus, Pediatric knee joint, Finite element simulation, Biomechanics, Three-dimensional model

## Abstract

Three-dimensional models have been widely used to study knee joint biomechanics in both healthy and pathological conditions. However, the lack of data on pediatric knee models affected by a discoid lateral meniscus necessitates further investigation. This study analyzed the biomechanical behavior of a pediatric knee joint with a discoid lateral meniscus malformation and the effects of partial meniscectomy on restoring its normal configuration. The three-dimensional geometry was reconstructed from computed tomography and magnetic resonance imaging data to develop a finite element model of the pediatric knee. The finite element method was used to simulate the joint in an upright position, and contact, compressive, and shear stresses were analyzed across seven lateral meniscus configurations with varying residual tissue widths to simulate progressive degrees of partial meniscectomy. A discoid lateral meniscus altered knee biomechanics, increasing medial-compartment stress, associated with femoral cartilage damage. Under body weight loading, the pediatric model showed a significant rise in stress when the meniscal width fell below 12 mm. A residual meniscal width of 12 mm provided a more favorable biomechanical response in this pediatric knee model, potentially reducing cartilage damage and the risk of early degeneration after partial meniscectomy.

**Graphical abstract:**

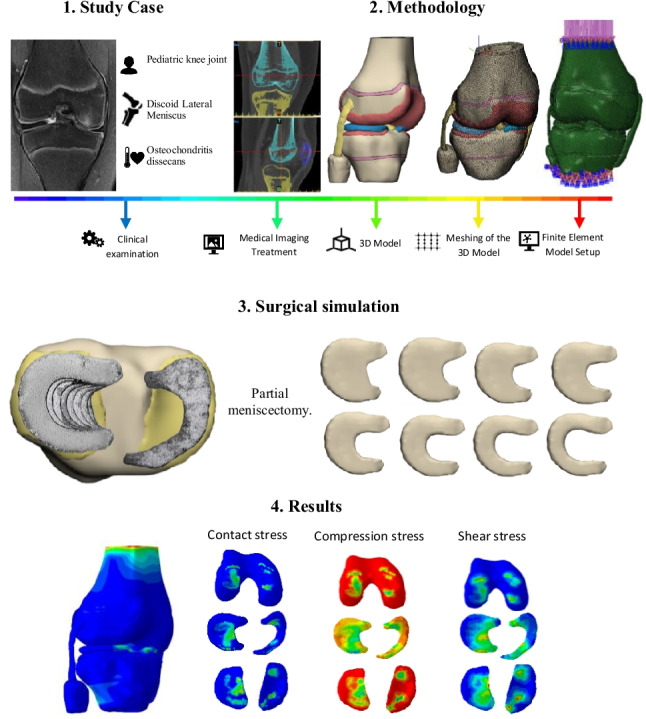

## Introduction

The discoid meniscus is a morphological [1] and structural [2–4] malformation of congenital origin [5–7] malformation that leads to a loss of the typical crescent shape of the meniscus, resulting in a partial or completely discoid configuration that occupies a larger surface area of the tibial cartilage. Menisci with this malformation are more prone to tears, degeneration, instability, and related symptoms such as pain, snapping, and limited extension [8–10]. This anomaly is more frequently observed in the lateral meniscus, with an incidence from 0.4% to 17%, compared to 0.1% to 0.3% in the medial meniscus [11, 12].

The importance of preserving meniscal function has been widely recognized since its role in knee joint biomechanics began to be systematically studied [4, 13]. Menisci play a crucial role in shock absorption, load distribution, joint lubrication, and maintaining optimal articulation between joint surfaces [14–16]. When the meniscal structure is compromised by pathological conditions such as a discoid lateral meniscus (DLM), the overall function of the knee joint is significantly affected. Furthermore, DLM has been suggested as a potential risk factor for osteochondritis dissecans (OCD) in young individuals, as it induces repetitive abnormal stress on the immature subchondral bone [17, 18].

Meniscal surgery aims to restore the physiological functions necessary for proper joint mechanics. However, surgical intervention may increase the risk of articular cartilage degeneration. As a result, partial meniscectomy and meniscal repair via suturing, both aimed at meniscus preservation and reshaping to restore a more physiological configuration, have become common therapeutic approaches for meniscal injuries [16]. Nonetheless, current treatment techniques and repair materials remain limited.

A critical aspect of these treatments is determining the optimal amount of meniscal tissue to be preserved, particularly in patients with DLM, where the lateral knee compartment exhibits distinct anatomical features, such as a flatter lateral femoral cartilage and a cup-shaped lateral tibial cartilage [13]. Regarding this issue, the scientific community presents two primary viewpoints: some experts recommend preserving a peripheral meniscal rim of 6–8 mm to prevent potential meniscal tears, others advocate for a rim of more than 10 mm to minimize excessive meniscal extrusion and reduce the risk of cartilage degeneration (Table 1).

**Table 1:** Summary of literature on residual meniscal width following partial meniscectomy

Arthroscopic technique	Researchers
Partial meniscectomy with a residual meniscal width between 6–8 mm	Hagino et al. [6] Ahn et al. [19] Lee et al. [20] Carabajal et al. [21] Ng et al. [22] Yang et al. [23] Nishino et al. [24] Hashimoto et al. [25]
Partial meniscectomy with a residual meniscal width greater than 10 mm	Tsujii et al. [26] Mochizuki et al. [27] Hashimoto et al. [28] Liu et al. [13] Yokoe et al. [29]]

The divergence of opinions regarding the optimal residual meniscal width stems from the potential risks the knee may face following surgery. Preserving a larger diameter of meniscal tissue increases the risk of meniscal tears [6, 20], whereas retaining a meniscal width of 6–8 mm may lead to osteochondral damage in cases of juvenile DLM [25], increased meniscal extrusion [24], and even axial misalignment and joint stability issues due to an inadequate meniscal width [30]. Gamble et al. [31] suggested that a 6–8 mm residual width is insufficient, recommending a minimum of 10 mm in children aged 8 years and at least 15 mm in adolescents.

Although extensive clinical studies have been conducted, current biomechanical research on this procedure remains limited, and numerical modeling is becoming an increasingly important tool for qualitative evaluation [32]. These models enable stress-state assessment and deformation analysis of the knee joint during routine activities [33, 34]. Finite element simulations using three-dimensional models provide insights into stress distribution within the knee joint following injuries, pathological degeneration, or malformations. They also play a crucial role in surgical treatment estimation, such as partial meniscectomy, overcoming the biomechanical constraints of traditional experiments under more objective conditions [35]. Therefore, there is a growing need to conduct such studies, as they replicate real-world problems and serve as powerful analytical tools, offering invaluable insights into real-life models [36].

**Fig. 1:**
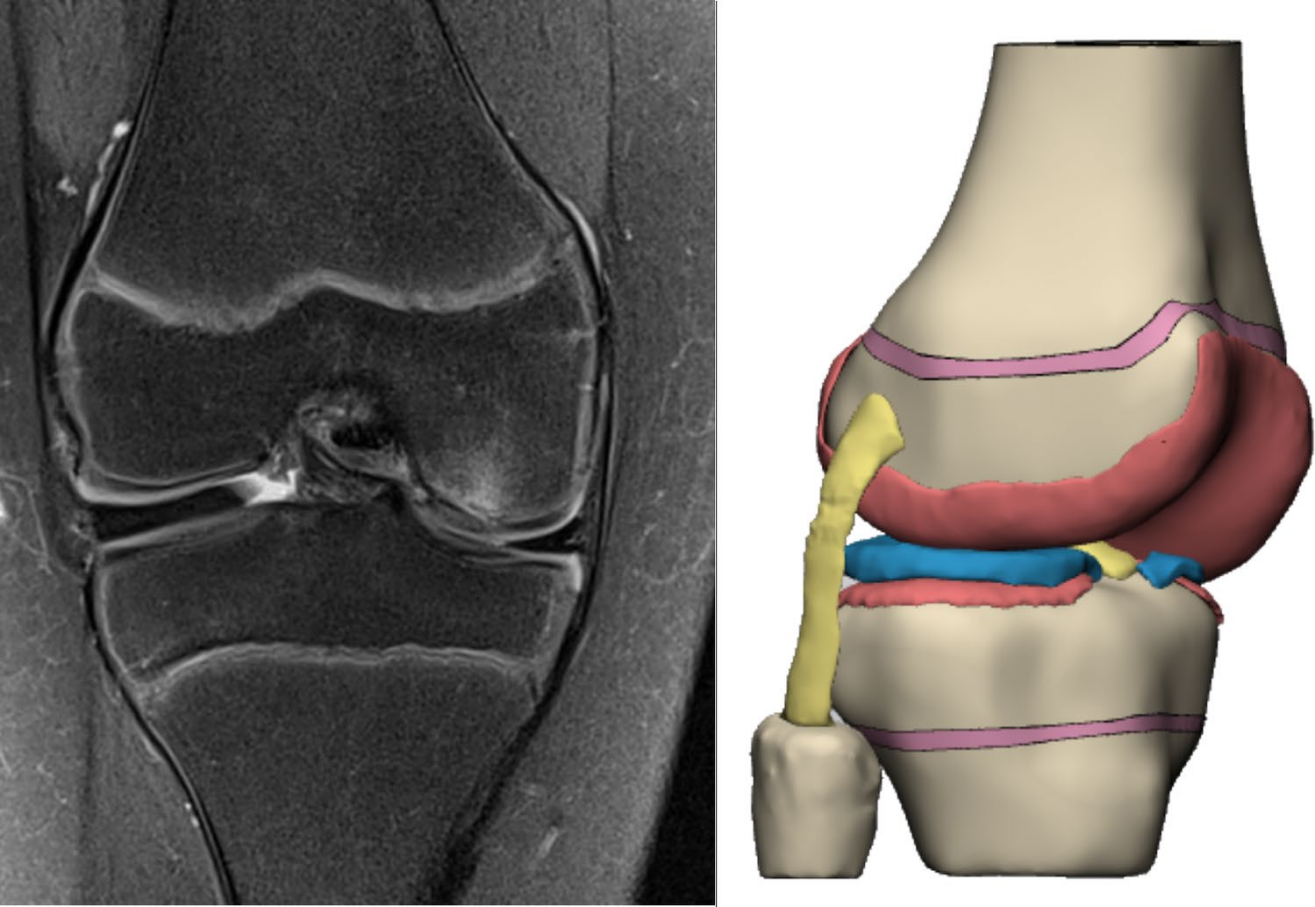
On the left, coronal view of the right knee showing discoid lateral meniscus (DLM) and associated chondral lesion. On the right, 3D model of the pediatric knee joint

Current technical literature lacks biomechanical studies of DLM in in pediatric knees. Such studies are critical to understand knee behavior during growth. The few pediatric reports available either provide no specific results on this malformation or rely on adult tissue properties [37, 38]. To address this gap, namely, the scarcity of finite element analyses To address this gap, specifically the limited availability of finite element analyses replicating the knee joint, particularly in pediatric structures, a comprehensive review of the existing literature on the mechanical properties of pediatric tissues was conducted. Because standardized pediatric experimental data are lacking and the reported values show considerable variability, a sensitivity analysis was carried out to determine and validate the mechanical property definitions used in the model. As a result, a pediatric finite element model was developed that incorporated femoral and tibial growth plates together with pediatric tissue properties [39–43], designed to evaluate a knee with a DLM and osteochondritis dissecans (OCD) (Fig. 1). This model serves as a reference for partial meniscectomy in a specific pediatric knee joint and emphasizes the key differences observed when compared with adult knees.

## Materials and methods

### 3D model of the knee joint

Computed Tomography (CT) and Magnetic Resonance Imaging (MRI) data were obtained from an 11-year-old patient weighing 54 kg. The patient had no underlying conditions but presented an osteochondral lesion in the femoral cartilage of the medial condyle. The patient was placed in a supine position, ensuring a non-weight-bearing condition during imaging. Medical images were imported into Materialise MIMICS 21.0 software to generate the 3D model of the knee joint. The bone structures were reconstructed from CT scans, comprising 298 medical images in DICOM format, each representing a 0.4 mm cross-sectional slice of the knee [32].

The articular cartilages, menisci, and ligaments (medial collateral, lateral collateral, anterior cruciate, and posterior cruciate) were segmented from MRI images. All segmentations were performed using a threshold-based segmentation approach, utilizing the Hounsfield unit (HU) scale to differentiate tissue types. The surfaces were smoothed using contour-editing tools to refine the 3D models, and Boolean operations were performed to ensure proper contact between components and eliminate geometric penetration.

**Fig. 2:**
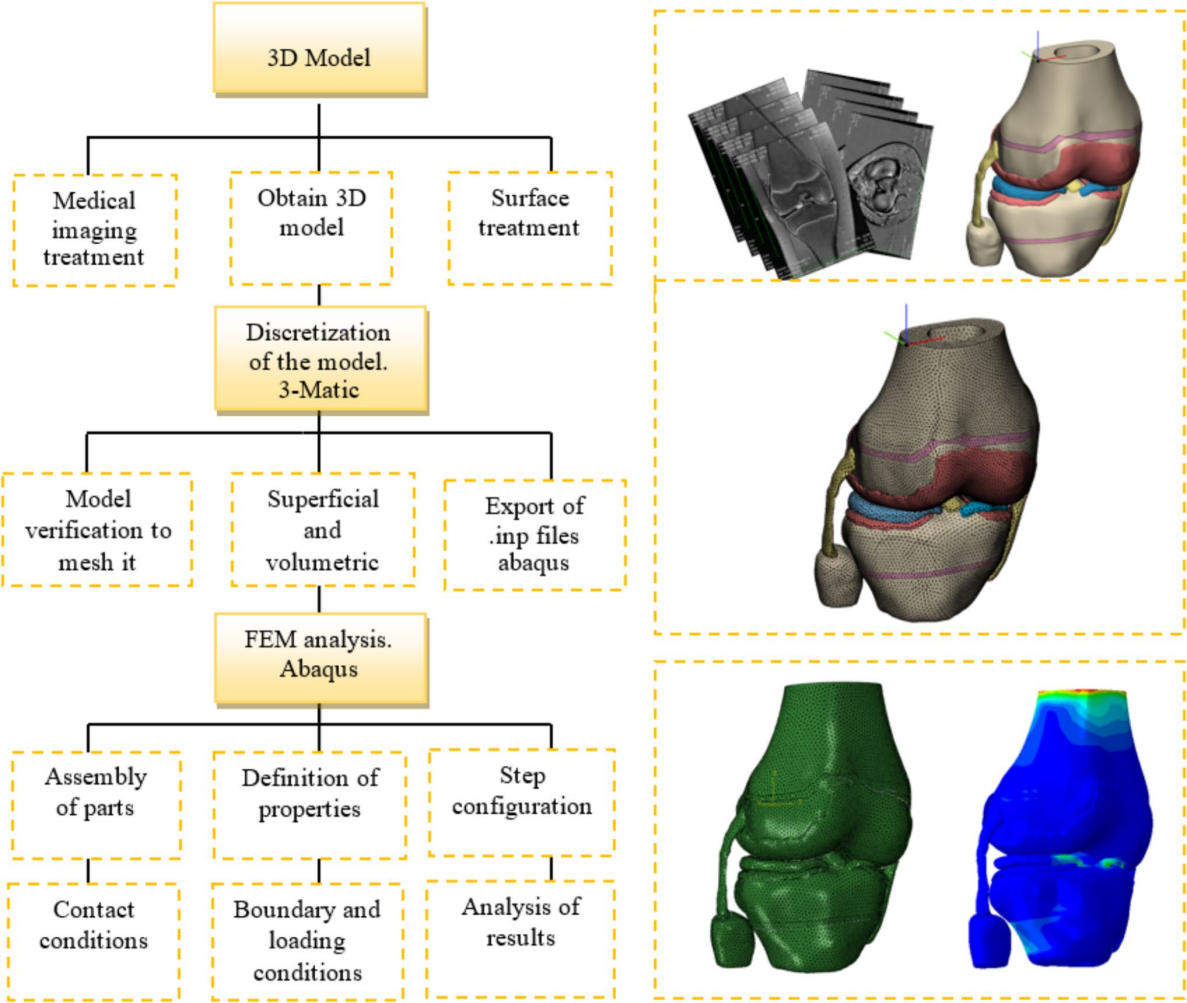
Work diagram for obtaining the three-dimensional model and finite element analysis

The assembled 3D knee joint model was then exported to Materialise 3-Matic 14.0 software, where surface quality verification was conducted. Each knee joint component was meshed with a homogeneous surface mesh, using a triangle length of 1.5 mm. This mesh size was validated through a mesh convergence analysis, showing result variations below 4%, indicating mesh insensitivity [36]. Figure 2 shows the flowchart for obtaining the finite element model for this study.

**Fig. 3:**
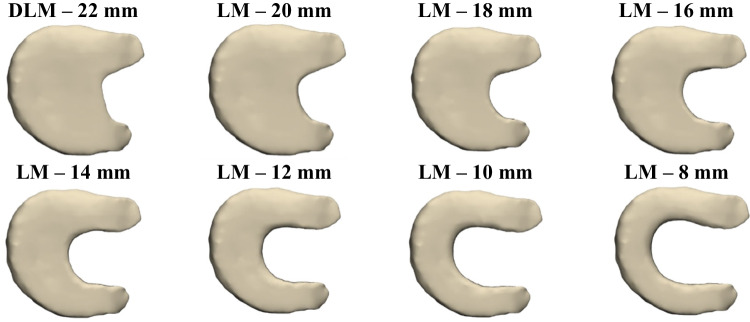
3D DLM model cut with different widths simulating partial meniscus meniscectomy

Volumetric meshes were generated for the bones, cartilages, and menisci, using four-node tetrahedral elements (C3D4). Ligaments were discretized with ten-node tetrahedral elements (C3D10MH) to better capture their nonlinear behavior [44–46]. A partial lateral meniscectomy was simulated by progressively reducing the meniscal width from 22 to 8 mm, generating seven meniscectomized models, each with a 2 mm decrement between successive models (Fig. 3).

### Pediatric finite element model

The meshed components were imported into Abaqus/CAE 2020 (Dassault Systèmes SIMULIA) where the 3D model assembly was performed. The mechanical properties of biological materials, contact conditions, loading scenarios, and motion constraints were defined to simulate the biomechanical behavior of the knee joint. The analysis was performed with the Abaqus/Standard solver in implicit mode. A general static analysis (Static, General) was conducted with geometric nonlinearity enabled (NLGEOM = ON). Increment control was set to automatic, following the default settings to ensure numerical stability.

#### Material properties

The bony structures were modeled as linearly elastic bodies [36], with a Young’s modulus (E) of 6000 MPa and a Poisson’s ratio (ν) of 0.3 [39–41]. The menisci and cartilages were assumed to be single-phase, linear isotropic materials, with E = 80 MPa and ν = 0.45 for the menisci, and E = 13 MPa and ν = 0.45 for the cartilages [38, 42]. The growth plates were assigned material properties of E = 6 MPa and ν = 0.48 [41]. The ligaments were defined as nonlinear elements and were modeled as nearly incompressible, transversely isotropic Neo-Hookean materials, whose strain energy function is expressed as follows [36, 44, 45, 47]:

1 $$\Phi ={C}_{1}\left(\overline{{G }_{1}}-3\right)+\frac{1}{{D}_{1}}{\left({J}_{F}-1\right)}^{2}+S(\lambda )$$

where $${C}_{1}$$ represents a bulk material constant related to shear modulus $$({C}_{1} =\raisebox{1ex}{$2$}\!\left/ \!\raisebox{-1ex}{$\mu $}\right.)$$. $${D}_{1}$$ is the inverse of modulus $$k / {C}_{1}=1000$$ [48], $${J}_{F}$$ represents the Jacobian matrix of deformation gradient, $$F$$ and $$\overline{{G }_{1}}$$ is the first invariant of the Cauchy-Green tensor and $$S(\lambda )$$ represents the strain energy function for the following conditions:

2 $$\lambda \frac{d S}{d \lambda }=\left\{\begin{array}{ccc}0& \lambda \le 1& \left(2a\right)\\ {C}_{3}\left({e}^{(\lambda -1){C}_{4}}-1\right)& 1\le \lambda \le {\lambda }^{*}& \left(2b\right)\\ {C}_{5}\lambda +{C}_{6}& {\lambda\geq\lambda }^{*}& \left(2c\right)\end{array}\right.$$

Under compression of $\lambda\le 1 (2a)$, the fibers did not support any compressive stresses. In the range $1 < \lambda < { \lambda }^{*} (2b)$, the stiffness of the fibers increased exponentially. For$\lambda \ge { \lambda }^{*} \left(2c\right)$, the fibers straightened and the stiffness increased linearly, ${C}_{3}$ represents the exponential stress scale, ${C}_{4}$ represents the rate of collagen without deformation, and ${C}_{5}$ represents the elastic modulus of stretched collagen fibers. To ensure stress continuity, the constant ${C}_{6}$ was introduced at${\lambda }^{*}$:

3 $${C}_{6}=\left({e}^{(\lambda^{*}-1){C}_{4}}-1\right){C}_{3}-\left({C}_{5}{\lambda }^{*}\right)$$

The values of the constants ${C}_{1}$, ${C}_{3}$, ${C}_{4}$, ${C}_{5}$ and ${C}_{6}$ are provided in Table 2.

**Table 2:** Material constants of the ligaments

	C_1_(MPa)	C_3_(MPa)	C_4_(-)	C_5_(MPa)	D_1_(MPa^−1^)	${\lambda }^{*}$(-)
ACL	1.95	0.0139	116.22	535.309	0.00683	1.046
PCL	3.25	0.1196	87.178	431.063	0.0041	1.035
MCL	1.44	0.57	48	467.1	0.00126	1.063
LCL	1.44	0.57	48	467.1	0.00126	1.063

*ACL* Anterior cruciate ligament; *PCL* Posterior cruciate ligament; *MCL* Medial collateral ligament; *LCL* Lateral collateral ligament

#### Contact and boundary conditions

The simulations were performed with the knee joint in full extension. Contact interactions were established between the femur and the femoral cartilage, the femoral cartilage and the menisci, the menisci and the tibial cartilage, and the tibial cartilage and the tibia [45, 49]. The contact conditions were defined such that all articular cartilages and ligament structures were rigidly attached to their respective bones; the same type of contact was applied between the growth plate and the trabecular bone. Between the articular cartilages and the menisci, a “surface-to-surface contact (Standard)” interaction was applied to ensure proper load transfer and mechanical behavior. Additionally, a frictionless normal contact property was assigned between the interacting surfaces to guarantee adequate transmission of forces.

The boundary conditions were defined to accurately replicate physiological constraints. The tibia and fibula were completely restricted in all degrees of freedom, preventing any displacement or rotation. In the femur, all rotational degrees of freedom were constrained except for rotation around the transverse axis, allowing for flexion and extension of the knee joint. Similarly, all translational degrees of freedom in the femur were restricted except for displacement along the longitudinal axis and the anteroposterior direction, permitting realistic knee motion [45].

#### Loading conditions

Partial meniscectomy in seven lateral meniscus configurations was evaluated under the following loading conditions. An initial axial preload of 100 N was applied to stabilize the system and induce passive ligament pretension without prescribing subject-specific initial strains. Subsequently, an axial load of 540 N was applied, corresponding to 100% of the body weight of the individual under study.

### Finite element model validation

The current literature reveals a marked scarcity of finite element biomechanical studies addressing the DLM in pediatric knees, and the few available reports generally rely on adult tissue properties. To overcome this limitation and ensure the mechanical validity of the analysis, a validation model was developed using adult tissue properties. The bone components were defined with E = 7300 MPa and ν = 0.3, the menisci with E = 120 MPa and ν = 0.45, and the cartilage with E = 15 MPa and ν = 0.475 [37, 38]. This model was designed to be benchmarked against previously published simulations of healthy adult knees, thereby verifying the accuracy and reliability of the finite element framework. In addition, a sensitivity analysis was conducted to assess the robustness of the model by varying the mechanical properties of the bone, cartilage, and meniscal tissues within the ranges reported for pediatric and adult populations. Different combinations of tissue stiffness and Poisson’s ratios were tested to evaluate how stress distributions changed between a joint modeled with adult tissue properties and one with pediatric tissue characteristics. This approach allowed us to confirm that the overall biomechanical trends observed in the pediatric configuration were consistent with those expected from adult knee models, reinforcing the reliability of the simulations and the validity of the comparative interpretation. This methodological approach, consistent with prior pediatric knee studies [41, 43], provides a robust foundation supporting the present findings within the broader context of joint biomechanics research.

## Results

This section consists of two parts: a) Finite Element Model Validation: in which an initial validation of the finite element model was performed by simulating the finite element model with adult tissue mechanical properties. Given the limited availability of pediatric finite element models, the purpose of this section is to reproduce and validate the behavior of our finite element model against adult knee models previously reported in the literature, and b) Effects of DLM and Partial Meniscectomy in the Pediatric Model: in this section is analyzed presents the biomechanical behavior of a pediatric knee with a DLM and the effects of partial meniscectomy for restoring the normal configuration. For this purpose, the finite element model with pediatric tissue mechanical properties was used, including the femoral and tibial physes (growth plates). In all simulations, contact, compressive (min.principal), and shear (Tresca) stresses were quantified for each knee structure.

### Finite element model validation

**Fig. 4:**
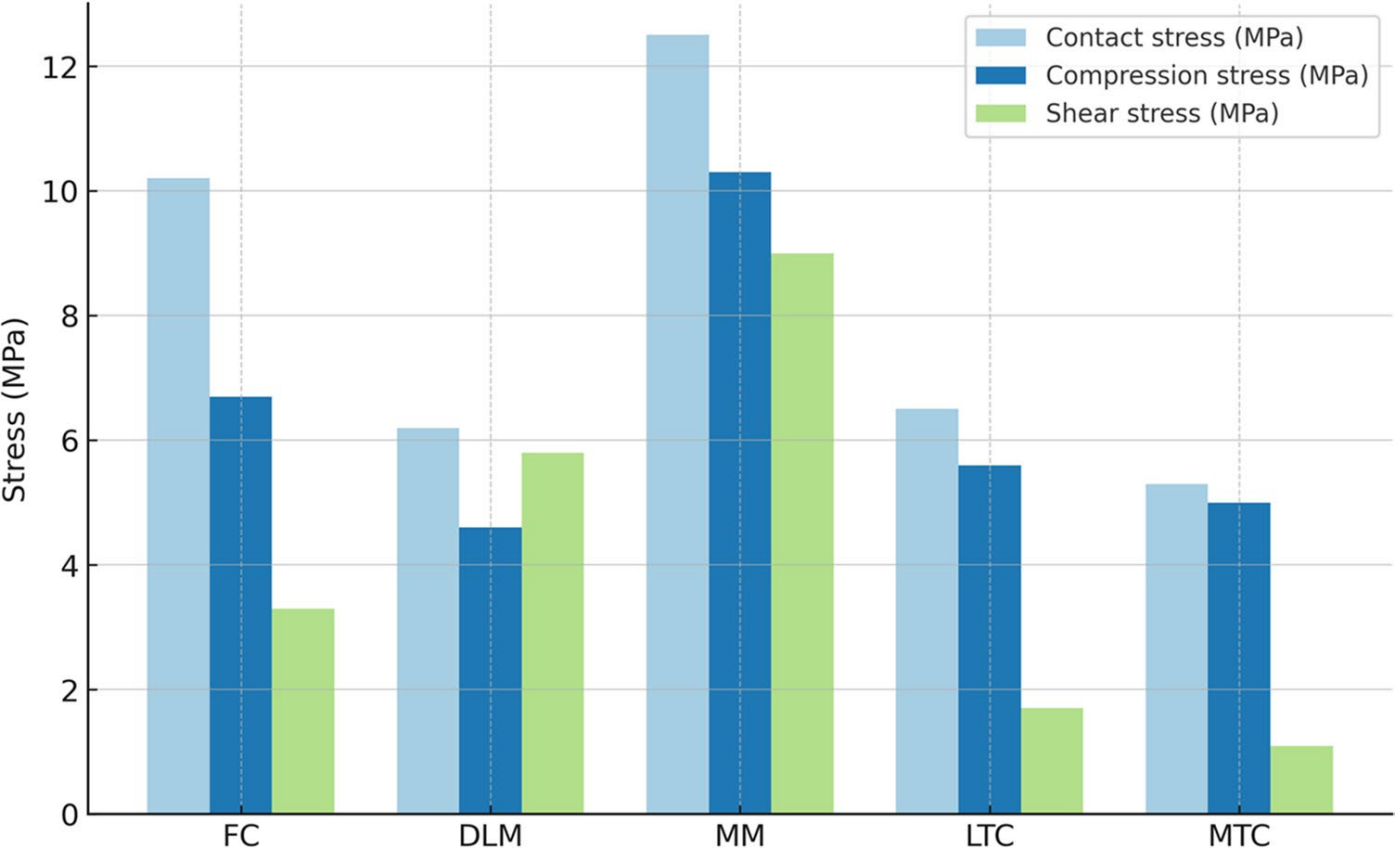
The results of contact, compressive, and shear stresses (MPa) obtained from the finite element validation model with a discoid lateral meniscus (DLM, 22 mm). FC (Femoral Cartilage), DLM (Discoid Lateral Meniscus), MM (Medial Meniscus), LTC (Lateral Tibial Cartilage), MTC (Medial Tibial Cartilage)

First, the finite element model of the knee was verified to ensure its mechanical validity and the model’s proper behavior under load, an essential requirement for interpreting the influence of the DLM. Given the scarcity of finite element studies on pediatric knees reported in the literature, the validation model was defined using adult tissue properties in order to compare the behavior of our model with existing simulations of healthy adult knees. The validated knee model exhibited a heterogeneous distribution of stresses across the articular surfaces, with a pronounced concentration in the medial compartment (Fig. 4). The medial meniscus showed the highest values across all three metrics, peak contact pressure (12.41 MPa), compressive stress (10.29 MPa), and shear stress (9 MPa), corroborating its primary role in transmitting and dissipating joint loads. Femoral cartilage reached intermediate magnitudes, peak contact pressure (10.23 MPa), compressive stress (6.65 MPa), and shear stress (3.36 MPa), whereas lateral structures, including the DLM, experienced comparatively lower stresses.

Collectively, these results demonstrate a consistent pattern across stress measures, with mechanical predominance of the medial compartment and a lesser contribution of lateral structures to load absorption and distribution. This distribution is consistent with results from knee models reported in the literature, in which the menisci support and redistribute most of the load, showing maximum stresses in the medial meniscus and the adjacent cartilage [36, 46].

### Effects of the DLM and partial meniscectomy in the pediatric model

**Fig. 5:**
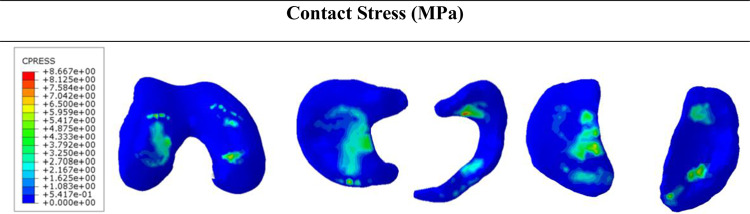
The results of contact stresses (MPa) obtained from the finite element pediatric model with a discoid lateral meniscus (DLM, 22 mm). From left to right: Femoral cartilage, B: Menisci, C: Tibial cartilage

First, the influence of the complete DLM (22 mm) on the biomechanics of the patient’s knee joint was analyzed. The contact stress results for DLM-22mm are presented in Fig. 5. The pediatric model showed that the peak contact stress was concentrated in the medial meniscus (8.67 MPa), followed by the lateral femoral cartilage (7.54 MPa) and the DLM (7.12 MPa). Lower stress levels were recorded in the remaining structures, with 6.74 MPa in the medial femoral cartilage, 6.86 MPa in the medial tibial cartilage and 6.56 in the lateral tibial cartilage.

**Fig. 6:**
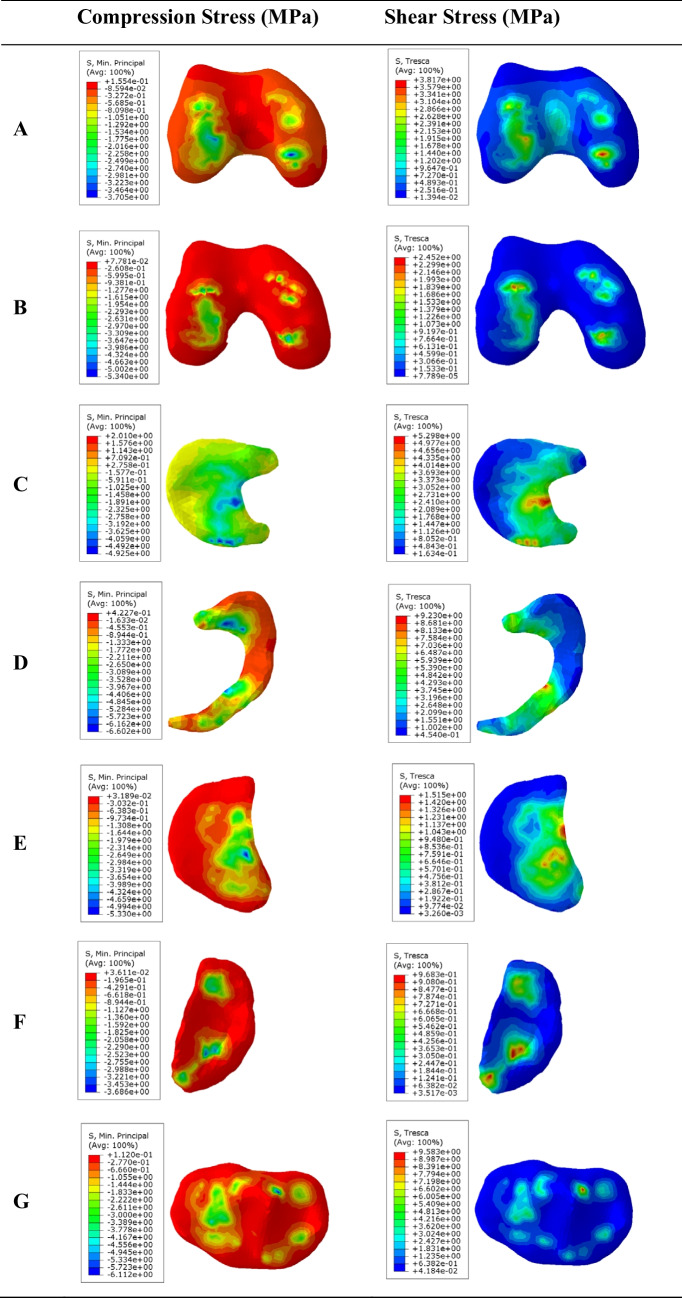
The results of compression stress (min. principal stress) left panel and shear stress (Tresca stress) right panel in discoid lateral meniscus (DLM-22 mm) for the pediatric model. From top to bottom: (**A**) Femur, (**B**) Femoral cartilage, (**C**) Lateral discoid meniscus (DLM), (**D**) Medial meniscus, (**E**) Lateral tibial cartilage, (**F**) Medial tibial cartilage, (**G**) Tibia

The compressive stress and shear stress results for DLM-22 mm are shown in Fig. 6. The peak stress was also located in the medial meniscus (6.60 MPa), followed by the femoral cartilage (5.34 MPa) and the lateral tibial cartilage (5.33 MPa), whereas the DLM (4.93 MPa) and the medial tibial cartilage (3.69 MPa) showed the lowest values. Overall, the pediatric model exhibited a more balanced stress distribution between the medial and lateral compartments. Regarding shear stress, the medial meniscus again showed the highest value (9.23 MPa), followed by the DLM (5.30 MPa) and the femoral cartilage (2.45 MPa), whereas the tibial cartilages remained low (1.52 MPa in the lateral and 0.97 MPa in the medial side).

When comparing the stress results obtained with the finite element model using the mechanical properties of adult tissue, employed for model validation (Fig. 4), and the finite element model defined with the properties of pediatric tissue and including the growth plate (Figs. 5 and 6), it was observed that contact, compression, and shear stresses were lower in the pediatric model, while the spatial distribution remained similar in both models, with the maximum load concentrated in the medial meniscus and, to a smaller degree, in DLM and tibial cartilages. This similarity suggests that global joint mechanics are preserved despite structural and material differences, as the pediatric model exhibits greater elasticity and stress-dissipation capacity due to lower tissue stiffness and the biomechanical influence of the growth plate, promoting a more uniform stress distribution while maintaining load transmission in the medial compartment.

**Fig. 7:**
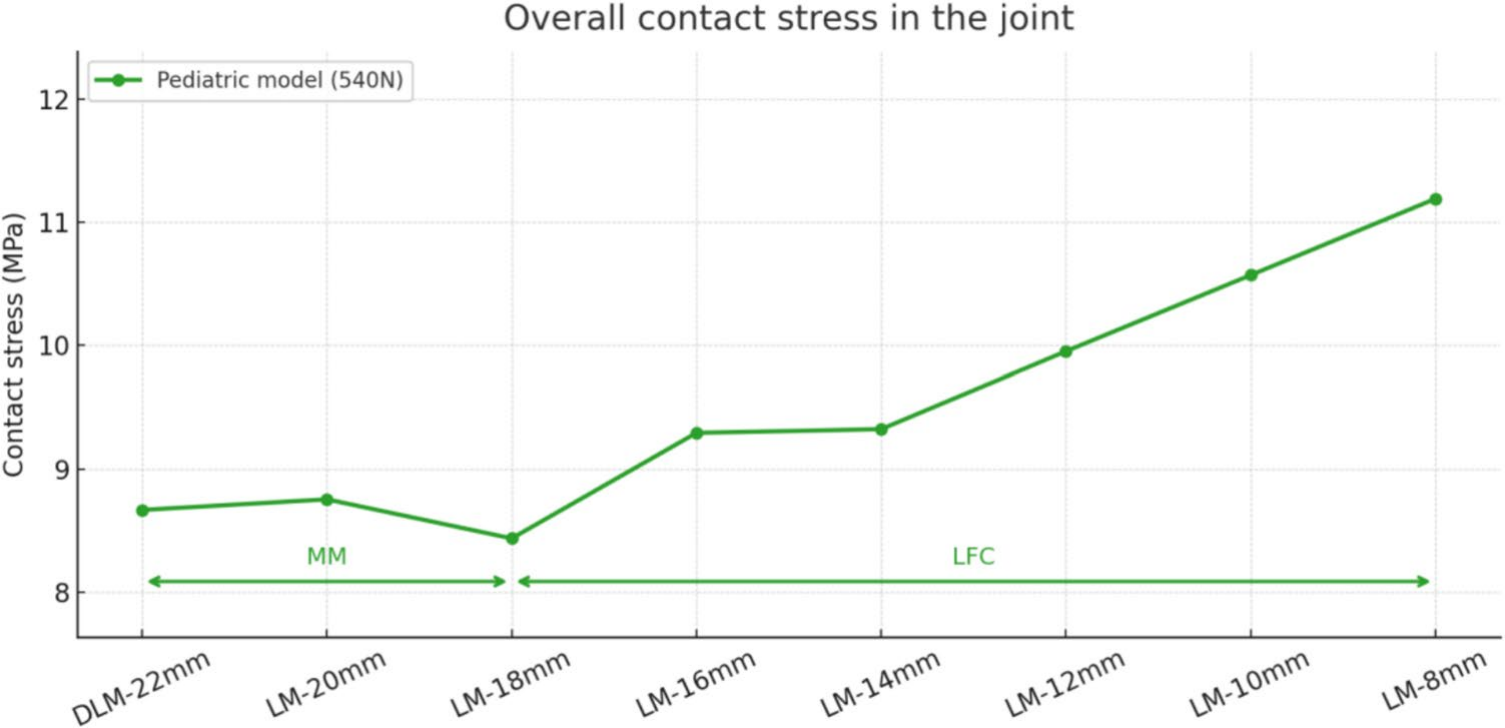
Results of the maximum contact stress in the knee joint for seven LM models. MM: medial meniscus; LFC: lateral femoral cartilage

Second, the stress distribution was analyzed in the seven models with different widths of residual meniscal tissue after partial meniscectomy, using the pediatric model. Figure 7 shows the maximum contact stress that occurs in the knee joint under an axial load of 540 N for each of the seven meniscal configurations.

The maximum contact stress remains practically constant at its minimum value when the residual width of the DLM is between 22 and 18 mm. Below 18 mm, the response changes qualitatively: the contact stress begins to increase progressively, and the location of the maximum stress shifts from the medial meniscus to the lateral femoral cartilage. Between 14 and 12 mm, a moderate increase of 7% is observed, and above 12 mm, the maximum stress exceeds 10 MPa at the lateral femoral cartilage, placing the load in a potentially damaging range for that compartment.

**Fig. 8:**
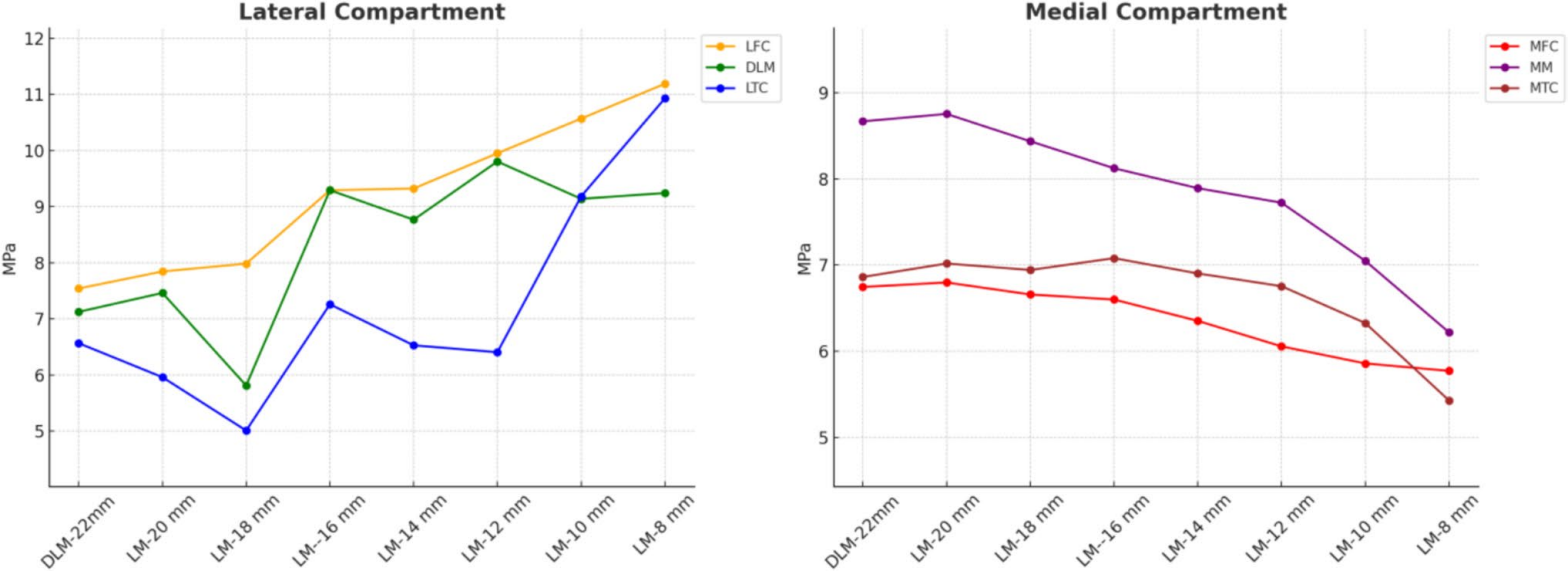
Results of the contact stress in the tissues of the lateral compartment (left panel) vs. the medial compartment (right panel). LFC (Lateral Femoral Cartilage), DLM (Discoid Lateral Meniscus), LTC (Lateral Tibial Cartilage), MFC (Medial Femoral Cartilage), MM (Medial Meniscus), MTC (Medial Tibial Cartilage)

The contact stress distribution across the different knee joint components for the seven meniscal models is shown in Fig. 8. The results indicate that the general trend of contact stress increased in lateral compartment tissues while decreasing in medial compartment tissues as the DLM width was reduced from 22 to 8 mm. Specifically, Stress increased in the lateral compartment tissues, with a 57% rise in the lateral meniscus and a 78% rise in the lateral tibial cartilage, whereas it decreased by 37% in the medial meniscus and by 19% in the medial tibial cartilage. The variation in femoral cartilage stresses was not substantial. Load redistribution remains consistent up to 12 mm, below 12 mm pronounced increases in stress are observed within the lateral compartment, consistent with a scenario that may compromise knee stability and joint health.

**Fig. 9:**
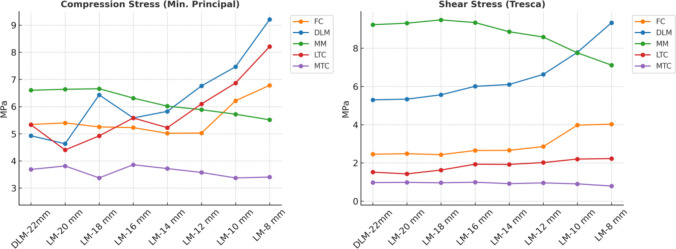
Results of compression stress (left panel) and shear stress (right panel) in the soft tissues of the knee joint for meniscectomy models. FC (Femoral Cartilage), DLM (Discoid Lateral Meniscus), MM (Medial Meniscus), LTC (Lateral Tibial Cartilage), MTC (Medial Tibial Cartilage)

The compressive stress results are shown in Fig. 9 (left panel). The general trend indicated that, as meniscal width decreased from 22 to 8 mm, compressive stress in the partially meniscectomized DLM increased from 4.92 MPa to 9.20 MPa, while stress in the medial meniscus decreased from 6.60 MPa to 5.51 MPa as the width decreased from 22 to 8 mm. In the femoral cartilage compressive stress remained stable until a 12 mm resection, at which point it reached a minimum value 5.025 MPa before increasing 6.78 MPa, at 8 mm width.

The shear stress (Tresca) results following surgery are shown in Fig. 9 (right panel). Shear stress in the femoral cartilage ranged from 2.45 to 4.03 MPa and rose sharply when the residual meniscal width fell below 12 mm. In the partially meniscectomized DLM, shear stress increased progressively, with a steeper rise below 12 mm, peaking at 9.33 MPa at 8 mm. In the medial meniscus, shear stress decreased from 9.23 to 7.11 MPa as width decreased from 22 to 8 mm. In the lateral tibial cartilage, shear stress increased from 1.51 to 2.20 MPa, whereas a mild reduction was observed in the medial tibial cartilage, from 0.97 to 0.79 MPa.

## Discussion

The malformation of DLM in the juvenile population, although uncommon, can have significant implications for knee joint biomechanics and contribute to pathological conditions such as cartilage degeneration, joint instability, and femoral condyle hypoplasia, among others. In this study, a case of an 11-year-old patient with DLM malformation and OCD in the medial femoral cartilage was analyzed using a 3D reconstruction of the patient’s knee joint and a detailed finite element analysis.

Previous studies have emphasized the value of finite element models as an effective tool for investigating knee biomechanics, aiding in both diagnosis and treatment planning [13, 29, 50]. These models provide a deeper understanding of the mechanical properties of biological tissues and generate insights that are challenging to obtain through experimental methods. However, their accuracy depends on precise geometric reconstruction and the implementation of a robust numerical model that ensures hypothesis validation and verification.

The results from the finite element analysis in this study suggest that DLM induces higher stress concentrations in the medial femoral cartilage, which may contribute to chondral lesion development, as also concluded in previous studies [7, 15, 17, 18]. As illustrated in Fig. 8, a progressive decrease in meniscal width, observed in the pediatric model leads to a marked increase in contact stress within the lateral compartment, while stress in the medial compartment tends to decline, so that the maximum contact stress shifts from the medial to the lateral compartment. This same pattern was also observed in compressive and shear stress (Fig. 9). The findings of this study, in terms of both the location and magnitude of peak stress, demonstrate strong agreement with prior finite element analyses of the knee joint [4, 13, 34, 43, 45, 46]. This consistency reinforces the reliability of the obtained data and validates the robustness of the computational model used.

The results of contact stress in the pediatric model, support that the redistribution of stresses associated with progressive meniscal narrowing may predispose the knee joint to cartilage degeneration, which is consistent with values previously reported in both pediatric and adult populations. Although a definitive clinical threshold for cartilage damage in the pediatric population has not been established, several studies suggest that contact stresses above 5 MPa may indicate high risk. For example, Dastgerdi et al. [41] reported stresses above this value during gait in pediatric models, possibly associated with cartilage overload. Similarly, in adult populations, Peña et al. [36] found contact stresses between 5 and 8 MPa in high-risk joint regions. In this study, the peak contact stress at the femoral condyle increased progressively with each resection, with a sharper rise below 12 mm. In the lateral tibial cartilage, stress increased markedly when the meniscal width fell below 12 mm. This behavior indicates load redistribution remains consistent up to a residual width of 12 mm, below that threshold further resections are associated with marked increases in stress within the lateral compartment, which may compromise knee stability and joint health.

With regard to compressive and shear stressesin the femoral cartilage and the lateral tibial cartilage remained nearly constant up to a 12 mm resection; beyond this threshold they increased progressively, with a steeper rise below 12 mm and peaking at 8 mm. These findings indicate that meniscal narrowing alters cartilage stress distribution under pediatric conditions, particularly when the residual width falls below 12 mm. DLM showed a progressive increase in compressive stress as meniscal width decreased, with a marked rise when the residual width fell below 12 mm. This behavior confirms that, even under physiological conditions, residual widths below 12 mm produce a significant increase in DLM stress, suggesting a high sensitivity of the pediatric lateral meniscus to changes in residual width following partial meniscectomy. In contrast, the medial meniscus exhibited the opposite pattern: stresses decreased progressively as lateral width was reduced, suggesting a compensatory mechanism of load redistribution toward the lateral compartment. In this context, not only the compressive stress is relevant but also the shear component, which, although less pronounced, intensified with larger resections. Because meniscal strength relies heavily on its ability to dissipate shear and circumferential stresses, a residual width below 12 mm significantly compromises its protective function.

In addition to analyzing compressive and shear stresses in the femoral cartilage, radial meniscal displacement was assessed as a potential indicator of immediate extrusion following meniscectomy. In models with meniscal widths ranging from 16 to 8 mm, medio-lateral displacement did not exceed 1 mm, remaining within the physiological range reported under static conditions [45, 51]. Although these findings do not indicate signs of immediate extrusion, several clinical studies have documented progressive extrusion during mid-term follow-up when the residual meniscal width is below 10 mm [24, 28]. This evidence supports the recommendation to preserve a meniscal width of at least 12 mm to maintain biomechanical function and reduce the risk of subsequent joint degeneration.

Determining the optimal meniscal width following surgery remains challenging due to the potential for postoperative knee damage. According to Hagino et al. [6] y Lee et al. [20], a greater meniscal tissue diameter increases the risk of meniscal tears. Hayashi et al. [52], reported a case of meniscal rupture 14 months post-surgery in a patient who had a 10 mm preserved meniscal width, supporting the recommendation of maintaining a post-surgical meniscal width between 6 and 8 mm. However, preserving this width range may accelerate osteochondral damage in cases of juvenile DLM [25], increase meniscal extrusion [24], and contribute to axial misalignment and joint instability [30].

For a peripheral width between 6 and 8 mm, Hagino et al. [6] reported favorable short-term outcomes, but noted that long-term follow-up revealed degenerative changes and lower limb alignment alterations. Similarly, Lee et al. [20] found that more than 30% of patients exhibited unfavorable clinical outcomes during an average 10-year follow-up, with progressive femoral cartilage degeneration. On the other hand, studies focusing on arthroscopic techniques with meniscal widths greater than 10 mm have demonstrated positive postoperative results. Hashimoto et al. [28] highlighted significant benefits, such as the absence of pain and meniscal extrusion, after two years of follow-up. Likewise, Mochizuki et al. [27] identified younger patient age and smaller preserved meniscal width as predictive factors for cartilage degeneration after DLM surgery. These findings have led to a reconsideration of the traditionally recommended surgical width of 6–8 mm [53].

The finite element simulations in this study revealed two key findings. First, preserving a partially meniscectomized DLM width greater than 12 mm does not provide a significant biomechanical advantage under a moderate 540 N load, this was also reported by Liu et al. [13]. Second, when the meniscal width is reduced below this threshold, the biomechanical environment of the knee deteriorates considerably. In this particular case, the finding supports and extends existing evidence on the influence of partially meniscectomized DLM width on pediatric knee biomechanics and is consistent with clinical studies in juvenile populations that recommend preserving a meniscal width greater than 10 mm [31].

Our results highlight several key aspects. First, as the peripheral width of the meniscus decreased, the location of peak stress shifted from the medial compartment to the lateral compartment, consistent with previous findings [13] [29]. Second, contact stress in the femoral cartilage remained below the failure thresholds reported in the literature [46, 54, 55]. During this period, femoral cartilage and surrounding structures have not yet reached full biomechanical maturity, which may compromise their ability to withstand high mechanical stresses and increase the risk of structural damage or growth-related alterations. The variation in stress distribution within the medial tibial cartilage was not significant, whereas a substantial increase in stress within the lateral tibial cartilage was observed when the peripheral meniscal width was reduced below 12 mm. This increased stress in the lateral tibial cartilage suggests a higher likelihood of developing post-surgical osteoarthritis in this region. The results demonstrate that post-surgical conditions in the operated compartment directly influence lateral compartment biomechanics, further reinforcing the need for careful surgical planning in cases of pediatric DLM.

This study has some limitations. First, it examines a single pediatric case, selected due to limited availability of clinical cases given the rarity of lateral discoid meniscus with osteochondritis dissecans and the low incidence of lateral discoid meniscus in children (~ 5%). Given the scarcity of pediatric finite element studies, this case provides detailed biomechanical evidence but does not support broad generalization. The results could be further strengthened by analyzing a larger number of pediatric knee models, which would allow for a more comprehensive statistical analysis. Second, the present analysis was performed in a static position; the study of knee dynamics remains a future line of research. In addition, the lack of long-term follow-up and postoperative clinical evaluations limits the ability to assess how joint biomechanics evolve with the child’s growth and development. Although this study does not include clinical follow-up, future validations could rely on advanced MRI and functional questionnaires to correlate biomechanical predictions with actual cartilage outcomes after meniscectomy.

The limited availability of pediatric tissue data made material assignment a challenge. Our model, based on a pediatric knee, addressed this limitation with a broad sensitivity analysis. Different combinations of bone and soft tissue properties were tested within the ranges reported for children. The results obtained using pediatric tissue properties followed a trend consistent with that reported in previous studies of adult knees, supporting the biomechanical validity of the pediatric model used and suggesting that, for this case study, the fundamental biomechanical mechanisms are aligned with those observed in adult knee models reported in the literature.

## Conclusions

This study highlights that DLM malformation disrupts knee biomechanics by increasing stress concentration in the medial compartment, which is linked to cartilage damage in the femoral condyle. Partial meniscectomy modifies this pattern by redistributing stresses between compartments, but when the meniscal width is reduced to less than 12 mm, overload shifts to the lateral tibial cartilage, raising the risk of secondary injury.

This study provides a novel perspective by focusing specifically on pediatric knee biomechanics, an area still underexplored compared to the extensive finite element studies in adult knees. The pediatric model identified safe meniscal width ranges in growing knees: under body weight loading, widths below 12 mm compromise stability. These findings emphasize that maintaining a width of at least 12 mm is biomechanically safer for this study case, establish for the first time a link between stress distribution and MRI detected lesions such as OCD, and support the importance of early detection and personalized treatment in the pediatric population.

The pediatric knee covers a wide age range and presents varying degrees of skeletal maturity. In addition, the heterogeneity in the characteristics of DLM related lesions further supports the recommendation for a personalized treatment approach, involving an individualized assessment of each case prior to surgical intervention.

## Data Availability

The datasets generated and/or analyzed during the current study are available from the corresponding author upon reasonable request or are included in this published article.
